# Mitral and Tufted Cells Are Potential Cellular Targets of Nitration in the Olfactory Bulb of Aged Mice

**DOI:** 10.1371/journal.pone.0059673

**Published:** 2013-03-18

**Authors:** Myung Jae Yang, Sooyeon Sim, Ji Hyun Jeon, Eojin Jeong, Hyoung-Chin Kim, Yong-Jin Park, In-Beom Kim

**Affiliations:** 1 Department of Otolaryngology-Head and Neck Surgery, College of Medicine, The Catholic University of Korea, St. Vincent's Hospital, Suwon, Gyeonggi-do, Korea; 2 Department of Anatomy, College of Medicine, The Catholic University of Korea, Seoul, Korea; 3 Biomedical Mouse Resource Center, Ochang Branch, Korea Research Institute of Bioscience and Biotechnology, Ochang-eup, Chungcheongbuk-do, Korea; The University of Queensland, Australia

## Abstract

Olfactory sensory function declines with age; though, the underlying molecular changes that occur in the olfactory bulb (OB) are relatively unknown. An important cellular signaling molecule involved in the processing, modulation, and formation of olfactory memories is nitric oxide (NO). However, excess NO can result in the production of peroxynitrite to cause oxidative and nitrosative stress. In this study, we assessed whether changes in the expression of 3-nitrotyrosine (3-NT), a neurochemical marker of peroxynitrite and thus oxidative damage, exists in the OB of young, adult, middle-aged, and aged mice. Our results demonstrate that OB 3-NT levels increase with age in normal C57BL/6 mice. Moreover, in aged mice, 3-NT immunoreactivity was found in some blood vessels and microglia throughout the OB. Notably, large and strongly immunoreactive puncta were found in mitral and tufted cells, and these were identified as lipofuscin granules. Additionally, we found many small-labeled puncta within the glomeruli of the glomerular layer and in the external plexiform layer, and these were localized to mitochondria and discrete segments of mitral and tufted dendritic plasma membranes. These results suggest that mitral and tufted cells are potential cellular targets of nitration, along with microglia and blood vessels, in the OB during aging.

## Introduction

The olfactory bulb (OB) is the first relay station in odor information processing such as acuity, discrimination, and memory [1]–[3]. In addition, the OB has a laminar organization of well-defined neuron types, contains a variety of neurotransmitters and their receptors, and has a unique capacity for synaptic plasticity. Thus, the OB has been widely used as a model system to study brain development, including adult neurogenesis and synaptic mechanisms in long-term potentiation and olfactory processing [4]–[8].

It has been reported that olfactory sensory function, including our sense of smell and our ability to discriminate between smells declines with age [9]. Indeed, it has been found that more than 75% of people over the age of 80 years demonstrate evidence of olfactory impairment [9]. Moreover, olfactory dysfunctions are common in neurodegenerative disorders, including Alzheimer's disease and Parkinson's disease [10], [11], in which cholinergic and dopaminergic modulatory systems, respectively, are affected in elderly patients [12], [13]. However, our knowledge of the cellular and molecular changes that occur during normal aging in order to bring about such decline in OB processing is relatively limited.

Among aging theories, the free radical theory proposes that aging is the cumulative result of oxidative damage to DNA, lipids, and proteins [14], [15]. Nitric oxide (NO), an important source of free radicals, is a diffusible gas that acts as a neuromodulator. Generated by NO synthase, it is involved in various physiological and pathological processes, such as synaptic plasticity, neurodegeneration, and aging in the brain [16]–[19]. Toxicity occurs when excess NO rapidly combines with another free radical, superoxide, to form the powerful oxidizing and nitrating agent, peroxynitrite. The selective nitration of protein tyrosine residues by peroxynitrite causes cellular dysfunction, DNA damage, and cell death, as well as the formation of 3-nitrotyrosine (3-NT) [20]–[22]. For this reason, 3-NT is considered a “footprint” of nitric oxide generation, as well as a neurochemical marker for oxidative damage. Interestingly, it has been reported that 3-NT levels increase during aging. Indeed, several recent studies on the role of free radicals in aging have demonstrated that 3-NT levels are increased in the cerebral cortex, hippocampus [23]–[27], and cerebellum [25], [28], [29] of aged rats. Moreover, oxidative protein damage has been associated with sensory dysfunction in aging [30], [31], [32], [33]. For example, Vaishnav et al. [34] reported that 3-NT levels are increased in the OB of 20-month-old mice. However, age-related changes in the cellular and subcellular localization of 3-NT, and its relationship with nitric oxide synthase (NOS) isoforms, the main sources of NO, remain unclear.

In the present study, we aimed to investigate age-related changes in 3-NT in the main OB of mice. The relationship between 3-NT formation and NOS isoforms was explored, and the cellular and subcellular localization of 3-NT in the OB was determined. Our findings indicate that 3-NT concentration is increased in the main OB with aging. Interestingly, we identified mitral and tufted cells as potential targets for protein nitration in the OB of aged mice.

## Results

### Age-related changes in 3-NT in the main OB of mice

**Figure 1:**
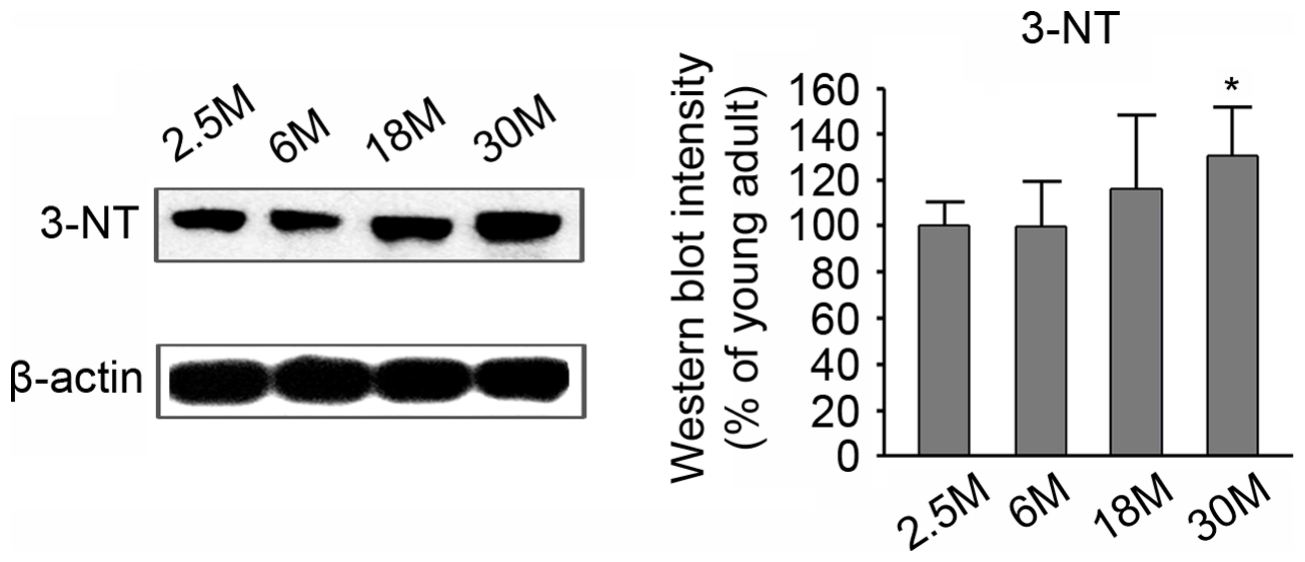
Age-related changes in 3-NT expression in the main OB of mice, as determined by western immunoblotting. Left panel shows the main 55-kDa band among multiple bands of representative blots for 3-NT. Right panel shows densitometric analyses of the 55-kDa-band intensity. Results are expressed as percentages (%) relative to the young (2.5 months old) mice. Data represent the mean ± SD values for 5 mice in each group. * *P*<0.05.

In order to investigate age-related changes in 3-NT in the main OB of mice, western immunoblotting was carried out on total protein extracts from the OB of young (2.5 months old), adult (6 months old), middle-aged (18 months old), and aged (30 months old) mice (Fig. 1A). As expected, multiple 3-NT-immunoreactive bands, ranging from ∼10 kDa to ∼200 kDa, were detected in all age groups. Among these bands, a representative 55 kDa band was demonstrated and quantitatively analyzed by densitometry (Fig. 1). The results demonstrate the degree of 3-NT formation as a function of age, with a statistically significant increase in 3-NT noted in the aged mice (*P*<0.05).

**Figure 2:**
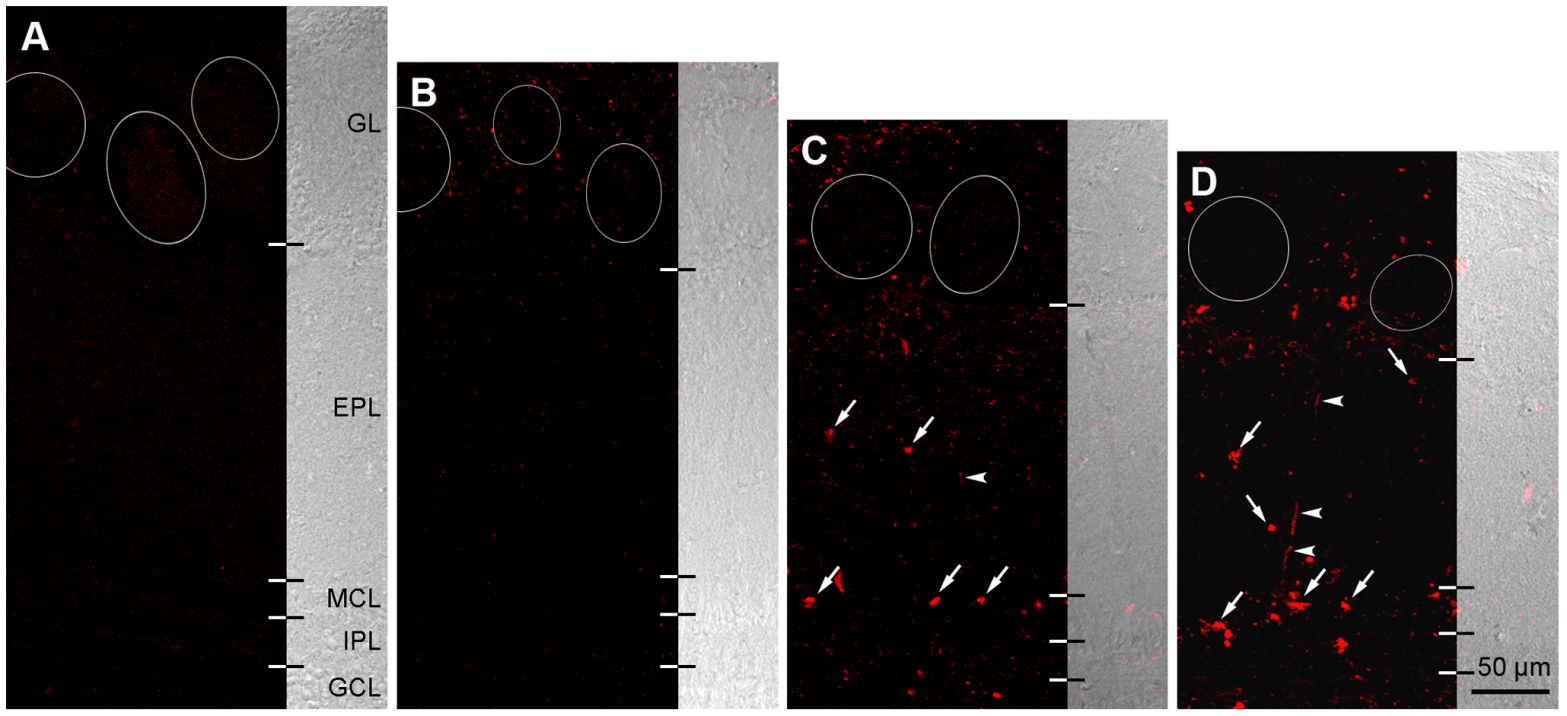
Age-related changes in 3-NT expression in the main OB of mice, as determined by immunohistochemistry. A: OB of young mouse (2.5 months old). 3-NT immunoreactivity is not detectable in any layers of the OB. EPL, external plexiform layer; GCL, granule cell layer; GL, glomerular layer; IPL, internal plexiform layer; MCL, mitral cell layer. B: OB of adult mouse (6 months old). Small weakly labeled puncta are visible in the GL. Within the GL, these puncta are frequently seen in the periglomerular region near glomeruli (circles). C: OB of middle-aged mouse (18 months old). Small 3-NT-immunoreactive puncta are found throughout all layers of the OB. Note the large strongly labeled puncta (arrows) located on the border of the MCL and EPL. Additionally, 3-NT-immunoreactive lines (arrowhead) are often seen in the EPL. D: OB of aged mouse (30 months old). The pattern of 3-NT immunoreactivity is similar to that of the middle-aged mouse, as shown in C. Large labeled puncta (arrows) and lines (arrowheads), which are observed in the MCL and the EPL, respectively, are found more frequently in the aged mouse compared to the middle-aged mouse.

We further examined age-related changes in 3-NT expression in the main OB of mice using immunohistochemistry (Fig. 2). Sparse 3-NT immunoreactivity was observed throughout the OB of young mice (Fig. 2A). In contrast, 3-NT immunolabeling was observed in adult mice, mainly in the periglomerular region of the glomerular layer (GL) in the form of small puncta (Fig. 2B). The expression of these 3-NT-immunoreactive puncta was further increased in the middle-aged mice and was observed throughout all layers of the main OB (Fig. 2C). Notably, large and intense immunoreactive puncta staining was found in the outer region of the mitral cell layer (MCL) and external plexiform layer (EPL) of this group (arrows in Fig. 2C). 3-NT expression in the main OB of aged mice was further increased in terms of intensity and distribution (Fig. 2D), especially in the MCL and EPL (arrows in Fig. 2D). Labeled radial lines (arrowheads in Fig. 2D) were also frequently observed in the EPL. Collectively, these results suggest that an age-related increase in 3-NT expression occurs in the main OB of mice.

### Relationship between 3-NT formation and expression of NOS isoforms in the OB with age

Since the main sources of NO are neuronal NOS (nNOS), endothelial NOS (eNOS), and inducible NOS (iNOS), we employed western immunoblotting to investigate which isoform of NOS was responsible for the increased 3-NT expression observed in the OB of aged mice.

**Figure 3:**
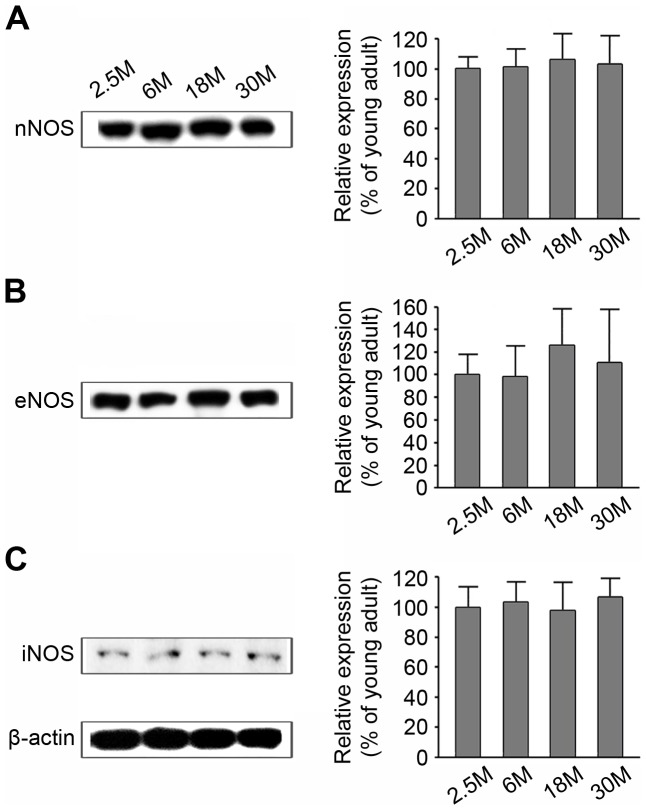
Age-related changes in NOS isoforms in the main OB of mice, as determined by western immunoblotting. A: nNOS. B: eNOS. C: iNOS. Left panel shows the representative immunoblots of the NOS isoforms. The three bands in the figure correspond to ∼155 kDa (A), ∼140 kDa (B), and ∼130 kDa (C), which are the molecular weights of nNOS, eNOS, and iNOS, respectively. The right panel shows the densitometric quantifications. Results are expressed as percentages (%) relative to the young mouse. Data represent the mean ± SD values of 5 mice in each group. No statistical differences were detected.

Western immunoblotting analysis of nNOS expression revealed a single ∼155 kD band across all age groups (Fig. 3A); however, no statistical difference between groups was observed. Similarly, in the case of eNOS, a single band at ∼140 kDa was detected in the OB of all groups (Fig. 3B), which displayed no significant between-group differences. Furthermore, a single band representing iNOS, at ∼130 kDa, was recognized in the OB of all age groups (Fig. 3C), and this also revealed no age-related differences. Collectively, these results suggest that no relationship exists between 3-NT formation with age and NOS expression in the main OB of mice.

### Cellular and subcellular localization of 3-NT in the main OB of aged mice

**Figure 4:**
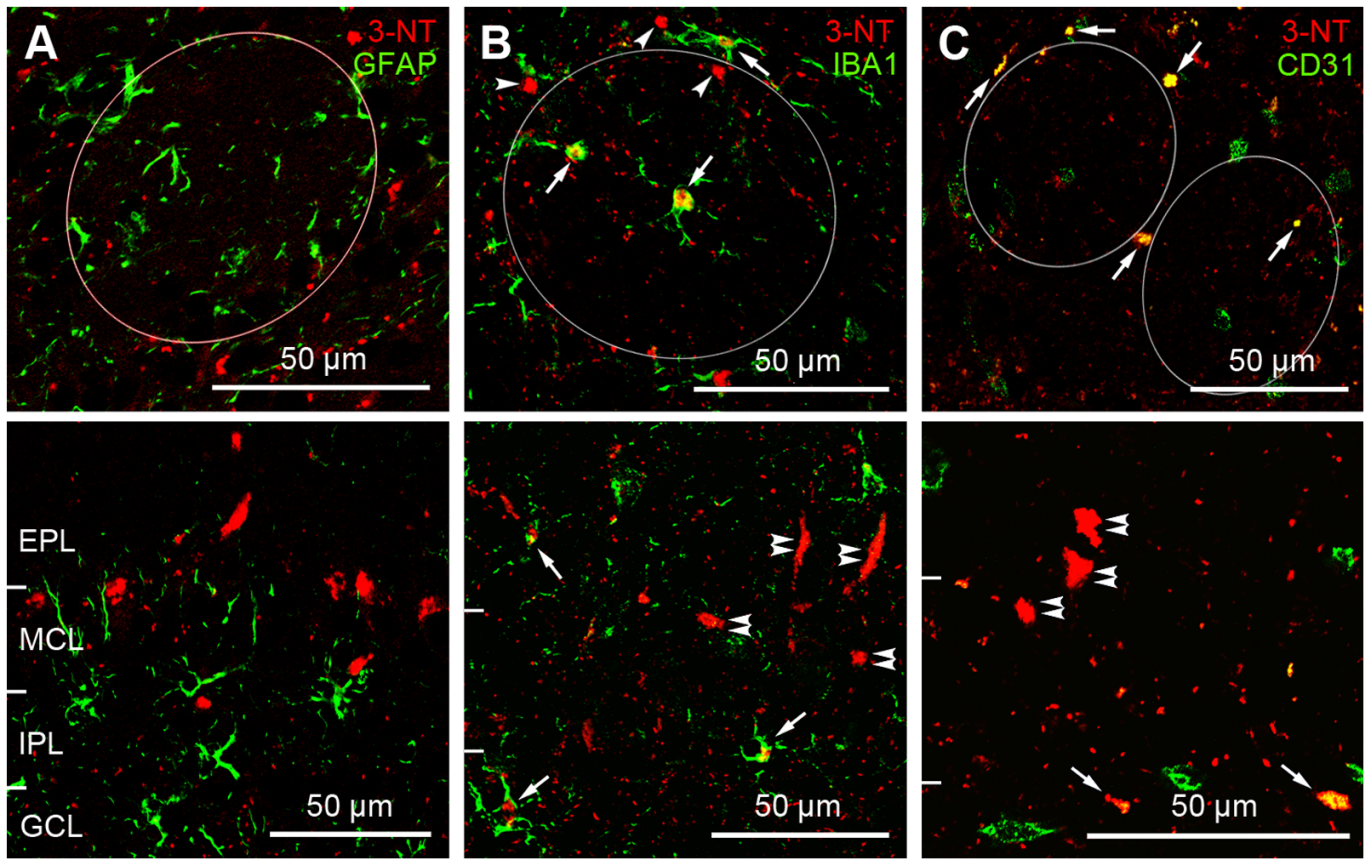
Cellular localization of 3-NT in the OB of aged mice by double-labeling immunohistochemistry with glial cell markers. Upper panels show the glomerular layer, while the lower panels show the mitral cell layer (MCL), the external plexiform layer (EPL) and internal plexiform layer (IPL). A: Merged image of 3-NT (red) and GFAP (green), an astrocyte marker. In both upper and lower panels, 3-NT does not colocalize with GFAP. GCL, granule cell layer. B: Merged image of 3-NT (red) and IBA1 (green), a microglial marker. In both upper and lower panels, some 3-NT puncta are localized within IBA1-labeled microglia (arrows), while other puncta within the glomerulus (circle) and in the periglomerular region do not show IBA1 immunoreactivity (arrowheads in upper panel). Note that large and strongly 3-NT-immunoreactive puncta (double arrowheads in lower panel) on the border of the MCL and EPL do not display IBA1 immunoreactivity. C: Merged image of 3-NT (red) and CD31 (green), an endothelial cell marker in blood vessels. In both upper and lower panels, several 3-NT puncta showing CD31 immunoreactivity (arrows) are seen, while the other 2 puncta localized within the glomerulus (right circle) and in the periglomerular region, respectively, do not show CD31 immunoreactivity (arrowheads in upper panel). Large and strongly labeled puncta (double arrowheads in lower panel) on the border of the MCL and EPL do not demonstrate CD31 immunoreactivity.

In the main OB of middle-aged and aged mice, 3-NT was expressed in various forms such as small and large puncta and radial-oriented lines (Figs. 2C and D). To determine the precise localization of 3-NT in aged mice, we first examined the colocalization of 3-NT and GFAP, an astrocytic marker [35] (Fig. 4A). However, we found no colocalization between 3-NT and GFAP, suggesting that 3-NT generated in the OB of aged mice was not localized to astrocytes.

In a previous report [34], 3-NT was found in microglia and blood vessels. However, in that study, the resolution of the figure was insufficient to clearly show the localization of 3-NT in microglia and blood vessels. We tried to confirm this report by conducting double-labeling experiments using anti-3-NT and anti-IBA1, a microglial marker [36] (Fig. 4B), or anti-CD31, a marker for blood vessel endothelial cells [37] (Fig. 4C). Our findings revealed some large 3-NT puncta colocalization with IBA1 within the glomerulus and in the periglomerular region of the GL; however, other large 3-NT puncta that were not colocalized with IBA1 were also observed. Additionally, the small 3-NT puncta displayed no colocalization with IBA1 (Fig. 4B). The same labeling pattern that was found in the GL was observed in the EPL, internal plexiform layer (IPL), and granule cell layer (GCL) (Fig. 4B). In addition, large 3-NT puncta in the outer region of the MCL, and long and thick-labeled radial-oriented lines within the EPL, showed no colocalization with IBA1 (Fig. 4B). Double-labeling with antibodies against 3-NT and CD31 showed similar results to double-labeling with anti-3-NT and IBA1 (Fig. 4C). Collectively, our findings suggest that some of the 3-NT generated in the OB of aged mice was localized to microglia and blood vessels.

**Figure 5:**
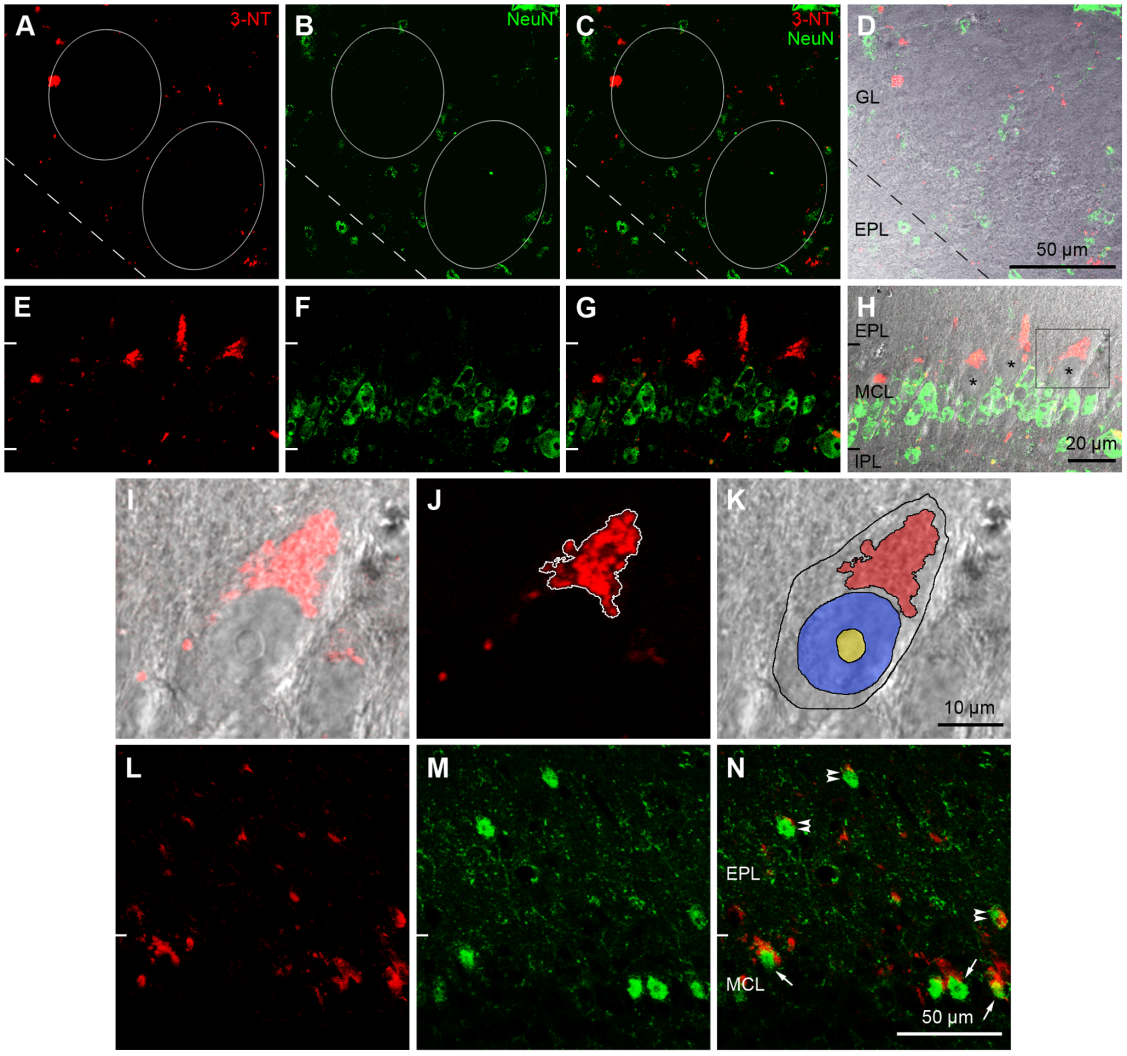
Cellular localization of 3-NT in the OB of aged mice by double-labeling immunohistochemistry with two neuronal markers, anti-NeuN (A–H) and anti-TBX21 (L–N). A–D: Glomerular layer. A: Small and large 3-NT-labeled puncta (red) are seen within the glomeruli (circles) and in the periglomerular region. B: Numerous periglomerular cells localized in the periglomerular region are labeled with anti-NeuN (green), a neuronal cell marker. C: In this merged image of A and B, few 3-NT labeled puncta are localized in NeuN-labeled periglomerular cells. D: A transmission light micrograph overlaid with C. E–H: Mitral cell layer (MCL) and 2 adjacent regions of the external plexiform layer (EPL) and the inner plexiform layer (IPL). E: Large 3-NT-labeled puncta are clearly seen on the border of the MCL and EPL. F: Many NeuN-labeled granule cells located in the MCL are observed. G: In this merged image of E and F, large 3-NT-labeled puncta are not localized to NeuN-labeled granule cells. H: In a transmission light micrograph overlaid with G, the large 3-NT-labeled puncta are exclusively localized externally to putative NeuN-negative mitral cells (asterisks). The rectangular area is magnified in I–K. I–K: A mitral cell. I: In a transmission light micrograph overlaid with red immunofluorescence, the 3-NT-labeled puncta with triangular shape is clearly seen in the apical portion of a putative mitral cell. J: In the confocal image using the red channel for 3-NT immunoreactivity, the labeled triangular puncta is outlined. K: A transmission light micrograph overlaid with the 3-NT puncta area (red) shown in J, a putative mitral cell (black outline), its nucleus (blue), and nucleolus (yellow). This image demonstrates that 3-NT is localized to the apical region of the mitral cell somata. IPL, inner plexiform layer. L–N: MCL and EPL. L: Similar to E, many large 3-NT-labeled puncta are observed in the MCL and EPL. M: Several somata located in the MCL and EPL are labeled with anti-TBX21 (green), a mitral and tufted cell marker. N: In this merged image of L and M, most of the large 3-NT labeled puncta (arrows) in the MCL are localized close to TBX21-labeled mitral cell somata. In the EPL, a few labeled puncta (double arrowheads) are localized close to TBX21-labeled tufted cell somata.

Next, we tested whether 3-NT is also localized to neurons in the main OB of aged mice. Double-labeling experiments were carried out using anti-3-NT and anti-NeuN, a neuronal marker [38] (Figs. 5A–K). As shown in Fig. 5A–H, there was no colocalization between 3-NT-labeled puncta and NeuN throughout all layers of the main OB. Of note, NeuN was not expressed in the OB mitral and tufted cells [38]. However, higher magnification images of a single 3-NT-labeled punctum in the outer MCL, overlaid with differential interference contrast images (Figs. 5I–K), strongly suggest that the large 3-NT-labeled puncta in the outer MCL were localized to the mitral cell somata, especially in the apical region from which the apical dendrite sprouts. In order to confirm the cellular identity of large 3-NT-labeled puncta in the MCL, we performed a double-labeling experiment with anti-3-NT and anti-TBX21, which is a newly introduced marker for OB mitral and tufted cells [39], [40] (Figs. 5L–N). We found that most of the large 3-NT-labeled puncta in the MCL were closely associated with TBX21-labeled mitral cells (arrows in Fig. 5N). With regard to the EPL, some of the large 3-NT-labeled puncta were closely associated with TBX21-labeled tufted cells (double arrowheads in Fig. 5N). These results suggest that mitral and tufted cells are potential targets of nitration in the OB of aging mice.

**Figure 6:**
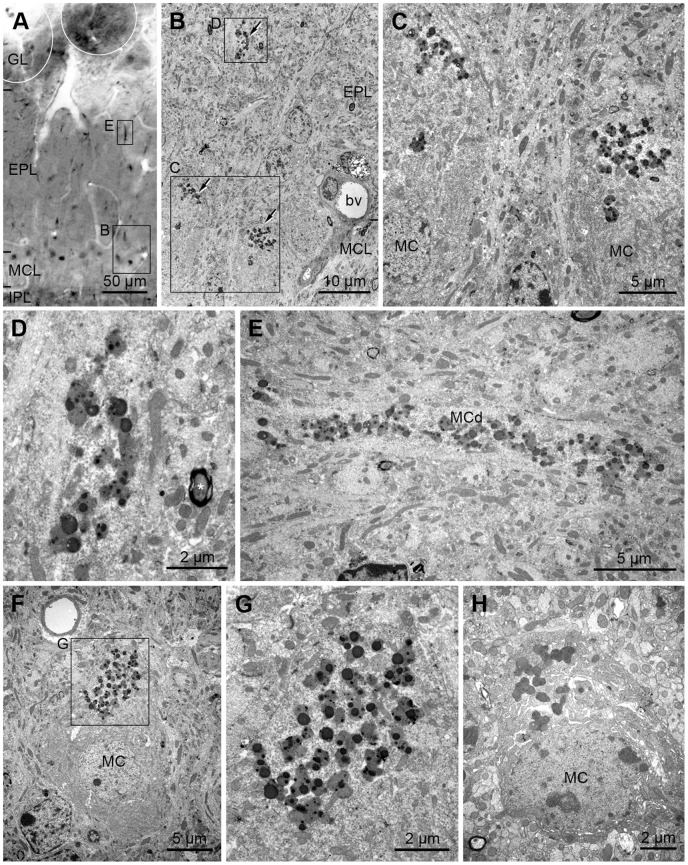
Identification and confirmation of the large 3-NT-labeled puncta located on the border of the mitral cell layer (MCL) and external plexiform layer (EPL) by electron microscopy. A: A light micrograph taken from a coronal section of a 30-month-old mouse OB processed for 3-NT immunoreactivity. 3-NT immunoreactivity is observed throughout all layers of the OB as small and large puncta. Large and strongly labeled puncta located on the border of the MCL and the EPL are clearly seen. Two rectangular regions are magnified in B and E. B: Three large labeled puncta (arrows) are identified as clusters composed of lipofuscin granules. One region, including 2 clusters on the border of the MCL and the EPL, and another region, including 1 cluster in the EPL are magnified in C and D, respectively. bv, blood vessel. C: Two labeled clusters are localized in the apical region of 2 mitral cell somata (MC). D: Higher magnification view of the upper rectangular region in B. 3-NT immunoreactivity is localized to the lipofuscin granules in the mitral cell dendrite. Asterisk indicates a myelinated axon. E: Higher magnification view of the rectangular region located in the EPL of A. Immunoreactivity is found in the lipofuscin granules within a mitral cell dendrite (MCd) running radially. F: Another electron micrograph showing an MC in an aged mouse OB; the MC had a typical triangular shape and contained 3-NT-labeled lipofuscin granules. Labeled lipofuscin granules are localized to the apical region of the MC. A rectangular region including a cluster of lipofuscin granules is magnified in G. G: A cluster of many 3-NT-labeled lipofuscin granules is clearly seen. H: An electron micrograph showing a mitral cell without 3-NT-labeled lipofuscin granules. The micrograph was taken from a coronal section of an aged mouse OB processed for 3-NT immunoreactivity without the incubation step with the primary antibody. No staining was observed in lipofuscin granules.

In order to further analyze the localization of the large 3-NT-labeled puncta in mitral and tufted cells, as well as the small 3-NT-labeled puncta observed throughout the main OB of aged mice, immunoelectron microscopy was performed. Figure 6 depicts the cellular and subcellular localization of 3-NT in mitral cells in the MCL of an aged mouse. 3-NT immunolabeling was exclusively found in lipofuscin granules within lysosomes located in the apical region of the mitral cell somata (Figs. 6B, C, and F, G). This location corresponds to the light microscopy findings shown in Figs. 5I–N and 6A. In addition, 3-NT-labeled radial-oriented lines in the EPL were identified as lipofuscin granules located in the mitral cell dendrites (Figs. 6B, D, and E). Collectively, these results indicate that large 3-NT-labeled puncta in the MCL and radial-oriented lines of the EPL are localized to lipofuscin granules in mitral cells.

**Figure 7:**
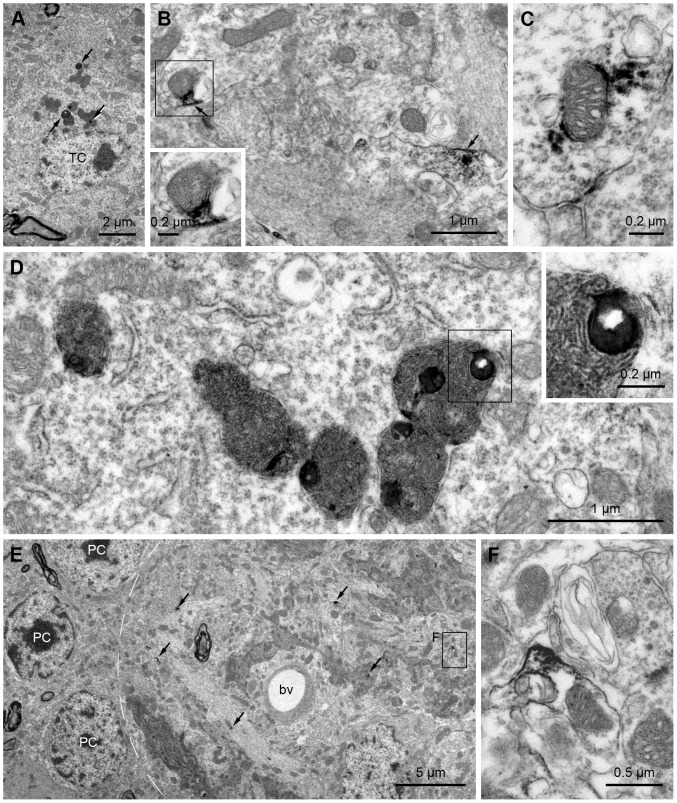
Identification and confirmation of the small 3-NT-labeled puncta in the external plexiform layer (EPL) and glomerular layer (GL) using electron microscopy. A–D: EPL. A: 3-NT-labeled lipofuscin granules are seen in a tufted cell soma (TC) located in the EPL. B: An electron micrograph showing the localization of the small 3-NT-labeled puncta in the EPL. 3-NT immunoreactivity is clearly seen in the discrete membranes (arrows) and a mitochondrion (rectangle) of the mitral cell dendrite. The rectangle is magnified in *inset*. C: Another example of a 3-NT-labeled mitochondrion of the mitral cell dendrite in the EPL. D: Several 3-NT-labeled lipofuscin granules are localized within putative autophagolysosomes. *Inset* magnifies the rectangular area of a putative autophagolysosome including a lipofuscin. Note that the putative autophagolysosome is filled with lamellated structures. E–F: GL. E: A low-power electron micrograph of the GL. Small 3-NT-labeled puncta (arrows) are scattered within a glomerulus (dotted line). A labeled puncta is magnified in F. bv, blood vessel; PC, periglomerular cell. F: Higher magnification view of the rectangular region of E. 3-NT immunoreactivity is localized to the mitochondrion and the discrete membrane of the neuropil.

In both the middle-aged and aged mice, many 3-NT-labeled puncta were observed in the GL and EPL (Figs. 2C and D). Figure 7 demonstrates the subcellular localization of these 3-NT-labeled puncta. Consistent with our light microscopy findings (Figs. 5L–N), 3-NT immunoreactivity of large puncta in the EPL was found in lipofuscin granules of tufted cell somata (Fig. 7A). Small 3-NT-labeled puncta in the GL and EPL were mainly localized to the discrete membranes of the neuropil and mitochondria of mitral and tufted cells (Figs. 7B–F). Interestingly, we often observed 3-NT-labeled lipofuscin granules localized within putative autophagolysosomes that were filled with lamellated structures, which appeared to be remnants of mitochondria (Fig. 7D). Taken together, these results highlight the possibility that proteins in the mitochondria of the mitral and tufted cell dendrites are the main targets of nitration in the aging OB.

## Discussion

In this study, we aimed to investigate age-related changes in 3-NT in the OB of mice, with specific regard for its cellular and subsubcellular localization. In doing so, we hoped to identify whether increased nitration in the aged brain acts as a contributing factor to the age-related decline in OB function. This study highlights the novel finding that 3-NT levels increase in mitral and tufted cells in the OB of aged mice, thus suggesting that enhanced nitration in these cells may play a potential role in age-related OB dysfunction.

NO is an important cellular signaling molecule involved in many physiological and pathological processes, including the processing, modulation, and formation of olfactory memories [41]–[44]. Indeed, the OB contains many intrinsic neurons that express nNOS [45]. High levels of NO are present under normal conditions, and it has been reported that these levels are increased by odor stimuli [46]. However, while NO is important for normal olfactory function, excessive levels of NO are potentially toxic and may make the OB more susceptible to oxidative stress, particularly to nitration, a form of protein oxidation. Interestingly, it has been reported that levels of 3-NT, which is considered a “footprint” of nitric oxide generation as well as a neurochemical marker for oxidative damage [47], is increased in the OB of 20-month-old mice [34].

In this study, we confirmed that 3-NT formation increases with age in the main OB. These results parallel age-related changes in 3-NT found in other brain regions, such as the cerebral cortex and hippocampus [23]–[27], [33], cerebellum [25], [28], [29], lateral geniculate nucleus [31], retina [30], and cochlea [32]. Using western immunoblot analyses, we demonstrated that the increased 3-NT observed with age in the main OB had no obvious correlation with an increased expression of the NOS isoforms, nNOS, eNOS, and iNOS. Similar findings to this have also been reported in other brain regions, including those mentioned above [25], [29], [48], [49]. Thus, the underlying mechanism responsible for the enhanced levels of NO and subsequent increased 3-NT formation, as observed in the aged OB of this study, remains to be determined; though one possibility is that NOS activity is increased independently of unchanged NOS protein levels. In addition, enhanced 3-NT levels with age may be due to the following: 1) a high level of proteins susceptible to nitration; 2) an increase in the activity of NADPH oxidase, as a source of superoxide; 3) reduced efficiency of antioxidative systems, i.e., SOD activity, which may decline or be downregulated in aging animals; and/or 4) age-related glutathione decrease in the OB, which is a known phenomenon in the aged OB [50]. Further studies are required to determine the precise mechanism by which 3-NT levels are increased.

3-NT has been identified in many diverse pathological conditions, such as atherosclerosis, pulmonary and heart disease, viral infections, and neurological disorders in which peroxynitrite induces neuronal cell dysfunction and death [51]–[53]. In this study, we observed that 3-NT immunoreactivity was localized to microglia and blood vessels. These findings are consistent with those found in the OB of humans diagnosed with Alzheimer's disease [54] and in aged rats [34], thus suggesting that nitration may lead to vascular dysfunction [53], [55], which in turn may lead to imbalances in ionic homeostasis in the OB, as in other brain regions [56], ultimately contributing to olfactory dysfunction, as proposed by Vaishnav et al. [34].

Lipofuscin is a chemically and morphologically polymorphous waste material. It is thought to be an end product of molecular damage to cell organelles as a result of oxygen free radicals, which accumulate in lysosomes, the primary site of waste disposal, in aging cells [57], [58]. Thus, lipofuscin has been considered a hallmark of aging [59]. In addition, it has been associated with numerous age-related diseases, including Alzheimer's disease and age-related macular degeneration [59]–[62]. Lipofuscin granules can contain toxic compounds, including A2E, which can lead to cellular dysfunction [58], [63]. In this study, 3-NT immunoreactivity was observed in mitochondria and lipofuscin granules. These subcellular localizations of 3-NT are consistent with those observed in the striatal neurons of the aged rat brain [64] and in hippocampal pyramidal neurons of aged humans [52].

Hirai et al. [65] demonstrated a close relationship between mitochondrial abnormalities and oxidative damage marked by 3-NT in hippocampal neurons in the brains of humans diagnosed with Alzheimer's disease. They also showed that abnormal mitochondria were associated with lipofuscin. In this study, 3-NT-immunoreactive lipofuscin granules were localized within putative autophagolysosomes filled with lamellated structures (Fig. 7), which appeared to originate from mitochondria. Autophagolysosomes are formed by the fusion of autophagosomes containing cellular waste, with lysosomes [66], [67]. Liposome fusion to mitochondria accounts for the prevalence of lipofuscin granules [68]. Based on recent findings that nitrated proteins can form insoluble aggregates [69] and that these aggregates result in the accumulation of lipofuscin [70], [71], our results highlight an intimate relationship among 3-NT formation, mitochondria, and lipofuscin granules in aged neurons, suggesting that the nitration of mitochondrial membrane proteins may contribute to lipofuscin granule development in the aging OB.

Recently, a very interesting report regarding age-related alterations in olfactory function was published by Richard et al. [72]. The authors demonstrated that a significant loss of synapses occurs in the OB with age without any anatomical alterations, including laminar organization and cell numbers; the loss of synapses disrupted the nature of the odor information passing through mitral and tufted cells, the main output neurons of the OB. Here, we call attention to the substantial localization of 3-NT to mitral and tufted cells observed in the OB of aged mice. In this study, we observed increased 3-NT expression in the somata and dendrites of mitral and tufted cells. Furthermore, we found increased subcellular 3-NT expressopm in lipofuscin granules, which also increase in mitral cells in an age-dependent manner [73]. Considering that protein nitration, as indicated by 3-NT-immunoreactivity, can directly cause cellular dysfunction [51], [53] and that lipofuscin may also lead to cellular dysfunction [58], [63], it is likely the mitral and tufted cells of the aged OB are functionally abnormal. Taken together, our findings suggest that age-related olfactory dysfunction is potentially attributable to alterations in mitral and tufted cells that occur as a result of nitrosative stress. However, future studies are needed to verify this.

In conclusion, the present study highlights the age-related changes that occur in the OB of mice. In particular, we found that the age-related increase in 3-NT was mainly localized to OB mitral and tufted cells, microglia, and blood vessels. Furthermore, 3-NT appeared to be exclusively associated with lipofuscin granules and mitochondria located within the somata and dendrites of mitral and tufted cells. This is the first time mitral and tufted cells have been implicated as potential cellular substrates for age-related olfactory dysfunction.

## Materials and Methods

### Ethical standards

This study was carried out in strict accordance with the recommendations provided in the Guide for the Care and Use of Laboratory Animals of the National Institutes of Health (NIH Publications No. 80–23) revised 1996. The protocol was approved by the IACUC (Institutional Animal Care and Use Committee) in the College of Medicine, The Catholic University of Korea (Approval Number: CUMS-2011-0083-01). All surgery was performed under ketamine and xylazine anesthesia, and all efforts were made to minimize suffering.

### Animals and tissue preservation

A total of 10 young (2.5 months old), 12 adult (6 months old), 15 middle-aged (18 months old), and 15 aged (30 months old) male C57BL/6 mice (Korea Research Institute of Bioscience and Biotechnology, Ochang, Korea) were used in this study. The mice were euthanized with 15% chloral hydrate. For western immunoblotting, animals were transcardially perfused with saline, and their brains were quickly removed. The main OB was then dissected on an ice-cold plate, frozen on dry ice, and stored at −70°C. For immunohistochemistry and immunoelectron microscopy, animals were transcardially perfused with saline and subsequently with 4% paraformaldehyde in 0.1M phosphate buffer (PB, pH 7.4). Brains were removed, post-fixed for 4 h in the same fixative, and then cryoprotected with 30% sucrose in 0.1 M PB. Finally, brains were embedded in Tissue-Tek (Sakura Finetechnical, Tokyo, Japan) and frozen with liquid nitrogen.

### Antibodies

Two different affinity-purified antibodies to 3-NT were used for western blotting: a mouse monoclonal antibody, that is, clone 2A12 (ab52309; Abcam, Cambridge, UK), and a rabbit polyclonal antibody (#06–284; Upstate Biotechnology, Lake Placid, NY). The monoclonal antibody detects multiple protein bands ranging from ∼10 kDa to ∼200 kDa on western immunoblots of mouse and rat brain lysates (manufacturer's technical information). Among these bands, a 55-kDa band has been found to be most prominent, as tested and verified in western immunoblots of rat kidney [74] and mouse lateral geniculate nucleus [75]. The polyclonal antibody detects multiple protein bands ranging from ∼16 kDa to ∼215 kDa, on western blots of A549 cells metabolically labeled with 3-NT (manufacturer's technical information). Of note, this antibody labeled many neurons in the cerebellum [28], auditory cortex [33] and lateral geniculate nucleus [31] of aged rats.

Antibodies against 3 isoforms of NOS were used for western immunoblot analyses. The goat polyclonal anti-nNOS antibody (ab1376; Abcam, Cambridge, MA) recognizes a single ∼155 kDa protein on western immunoblots of human muscle tissue lysate (manufacturer's technical information). The mouse monoclonal anti- eNOS antibody (#610296; BD Transduction Laboratories, Lexington, KY) detects a single band at ∼140 kDa in human endothelial cell lysates (manufacturer's technical information), as determined by immunoblots of the rat femoral artery [76]. A rabbit polyclonal anti-iNOS antibody (AB5382; Chemicon, International, Temecula, CA) detects a single band at ∼130 kDa for the rat liver (manufacturer's technical information), as determined by immunoblots of oligodendroglia isolated from rat brains stimulated with IFNγ/LPS [77].

In order to determine the localization of 3-NT in the OB of aged mice, we used the following published and characterized antibodies for double-labeling experiments: mouse monoclonal anti-glial fibrillary acidic protein (GFAP) antibody (MAB360; Millipore) as a marker for astrocytes [35]; rabbit polyclonal anti-ionized calcium binding adaptor molecule 1 (IBA1) antibody (Wako Pure Chemical Industries, Osaka, Japan) as a marker for microglial cells [36]; mouse monoclonal anti-CD31 antibody (#550274; BD Pharmingen, San Diego, CA) as a marker for endothelial cells on blood vessels [37], mouse monoclonal anti-NeuN antibody (MAB377; Millipore, Temecula, CA) as a marker for neurons [38]; and mouse monoclonal anti-T-bet/TBX21 (clone 4B10, 14–5825, eBioscience, San Diego, CA) as a marker for mitral and tufted cells [39], [40], [72], [78].

### Western immunoblotting

Western immunoblot analyses were performed on extracts of the main OB, which were homogenized in ice-cold RIPA buffer (50 mM Tris buffer, pH 8.0; 150 mM NaCl; 1% NP-40; 0.5% deoxycholate; and 0.1% SDS). Aliquots of tissue samples corresponding to 50 μg of total protein were heated at 100°C for 10 min with an equivalent volume of 2× sample buffer (containing 4% SDS and 10% mercaptoethanol) and loaded onto 6% polyacrylamide gels. The proteins were electrophoresed and blotted onto a nitrocellulose membrane. The membrane was blocked by treatment with 5% nonfat dry milk dissolved in 0.01M phosphate buffered saline (PBS, pH 7.4) containing 0.05% Tween-20 for 1 h at room temperature. The membrane was then incubated for 15 h at 4°C with anti-3-NT (dilution 1∶10,000), anti-nNOS (1∶1000), anti-eNOS (1∶2500), or anti-iNOS (1∶5000) in blocking solution. The membrane was rinsed 3 times with 0.05% Tween-20 in PBS, for 10 min each time, and incubated for 2 h at room temperature in a 1∶1,000 dilution of the appropriate biotin-conjugated IgG antibody (Vector Laboratories, Burlingame, CA). Afterwards, the membrane was rinsed 3 times with 0.05% Tween-20 in PBS, for 10 min each time, and incubated for 1 h at room temperature in ABC solution (Vector Laboratories). The blot was washed 3 times, for 10 min each time, and immunoreactive bands were detected using the Enhanced Chemiluminescence Detection Kit (Amersham, Arlington Heights, IL).

### Immunohistochemistry

Coronal cryo-sections of the main OB (40-µm thickness) were used. The sections were incubated in 10% normal donkey serum (NDS) and 1% Triton X-100 in PBS for 1 h at room temperature in order to block any nonspecific binding sites. They were then incubated with anti-3-NT (dilution 1∶500) in PBS containing 0.5% Triton X-100 for 1 day at 4°C. The sections were washed in PBS for 45 min (3×15 min), incubated in the presence of biotin-labeled donkey anti-rabbit IgG (Jackson Immuno Research, West Grove, PA; dilution 1∶100) for 2 h, and then washed 3 times in PBS for 45 min (3×15 min). The sections were subsequently incubated with Cy3-conjugated streptavidin in PBS (Jackson Immuno Research; dilution 1∶1000) for 1 h. After rinsing several times in PBS, the fluorescent specimens were mounted with Vectashield mounting media (Vector Laboratories, Burlingame, CA).

For double-labeling, coronal OB sections were incubated in 10% NDS and 1% Triton X-100 in PBS for 1 h at room temperature, and then incubated for 1 day in antibody mixtures composed of anti-3-NT and one of the antibodies against GFAP, IBA1, CD31, or NeuN. The sections were rinsed for 45 min (3×15 min) with PBS, and incubated in the presence of appropriate secondary antibodies conjugated with Cy3 (Jackson Immuno Research) and Alexa Fluor 488 (Molecular Probes, Eugene, OR) at a dilution of 1∶200 in PBS containing 0.5% Triton X-100 in PB at room temperature for 2 h. After rinsing several times in PBS, the fluorescent specimens were mounted with Vectashield. Digital images (1,024×1,024 pixels) were acquired using a Zeiss LSM 510 Meta confocal microscope (Carl Zeiss Co. Ltd., Jena, Germany), imported into Photoshop (Adobe Systems, San Jose, CA), and adjusted for brightness and contrast.

### Immunoelectron microscopy

Coronal vibratome sections of the main OB (40-µm thickness) were prepared. After blocking, the sections were incubated in a primary antibody solution at 4°C for 1 day, as used for light microscopy but without Triton X-100. The following immunocytochemical procedures were carried out at room temperature. The sections were washed in PBS for 45 min (3×15 min), incubated in biotin-labeled goat anti-mouse IgG (Jackson Immuno Research; dilution 1∶100) for 2 h, and then washed 3 times in PBS for 45 min (3×15 min). The sections were then incubated in ABC solution (Vector Laboratories) for 1 h, washed in 0.1 M Tris buffer (TB, pH 7.6), and preincubated in 3,3′-diaminobenzidine tetrahydrochloride (DAB) in TB for 10 min, followed by incubation in the same solution containing 0.05% hydrogen peroxide (H_2_O_2_) for an additional 10 min. The reaction was monitored using a low-power microscope and was stopped by replacing the DAB and H_2_O_2_ solution with TB.

Stained sections were post-fixed in 1% glutaraldehyde in PB for 1 h and, after washing in PB containing 4.5% sucrose for 15 min (3×5 min), they were post-fixed in 1% OsO_4_ in PB for 1 h. The sections were then rewashed in PB containing 4.5% sucrose and dehydrated in a graded series of alcohol. During the dehydration procedure, they were stained *en bloc* with 1% uranyl acetate in 70% alcohol for 1 h, transferred to propylene oxide, and flat-embedded in Epon 812. After curing at 60°C for 3 days, well-stained areas were cut out and attached to an Epon support for further ultrathin sectioning (Reichert-Jung, Nuβloch, Germany). Ultrathin sections (70–90 nm thickness) were collected on 1-hole grids coated with Formvar, and examined using an electron microscope (Jeol 1200EX, Tokyo, Japan).

### Statistical analysis

Data are represented as means ± standard deviation (SD). Statistical analysis was performed using ANOVA to evaluate the significance of differences between groups. *P* value<0.05 was considered statistically significant.
